# Examining the Relationship Between Free Will Belief and Job Crafting in Nurses

**DOI:** 10.1155/jonm/9991125

**Published:** 2025-09-10

**Authors:** Yue Wen, Song Wang

**Affiliations:** ^1^Department of Gastrointestinal Surgery, West China Hospital, Sichuan University/West China School of Nursing, Sichuan University, Chengdu, China; ^2^Department of Radiology, Huaxi MR Research Center (HMRRC), Institute of Radiology and Medical Imaging, West China Hospital, Sichuan University, Chengdu, China; ^3^Psychoradiology Key Laboratory of Sichuan Province, West China Hospital, Sichuan University, Chengdu, China

**Keywords:** belief in free will, big five personalities, Chinese nurses, job crafting

## Abstract

**Background:** Job crafting, whereby nurses proactively shape their roles, is critical for well-being and performance amidst demanding workloads. This study investigates the understudied role of belief in free will (BFW) as an antecedent to job crafting among nurses, employing two distinct methodologies to ensure robust findings.

**Methods:** Two studies were conducted with Chinese nurses. Study 1 (*N* = 274) used a cross-sectional design to examine the relation between BFW, as measured by the Free Will Scale, and job crafting, as assessed by the Measure of Job Crafting, controlling for Big Five personality traits via hierarchical regression analyses. Study 2 (*N* = 435) directly assessed BFW by classifying nurses as either free will or determinism believers using a philosophical prompt, and then compared job crafting scores via *t*-tests.

**Results:** Both studies converged to demonstrate a positive association of BFW with overall job crafting and its three dimensions (task, cognitive, and relational crafting). In Study 1, BFW significantly predicted overall job crafting as well as task and cognitive crafting, even after controlling for Big Five personality traits. Consistently, in Study 2, free will believers reported significantly higher levels of overall job crafting and its three dimensions compared to determinism believers.

**Conclusion:** These findings demonstrate that nurses' BFW is positively associated with their job crafting, suggesting that interventions that foster a sense of agency and control may empower nurses to proactively shape their jobs and improve their well-being. Further research should examine the generalizability of these findings across diverse populations and explore the underlying mechanisms linking BFW to job crafting.

## 1. Introduction

The modern landscape of nursing practice presents a complex interplay of demanding workloads, intricate patient needs, and rapidly evolving technological advancements, demanding not only specialized clinical skills but also a workforce that is actively engaged, remarkably adaptable, and profoundly resilient [1, 2]. In this challenging environment, the concept of job crafting, defined as the proactive and self-initiated behaviors that employees engage in to redesign their jobs to better align with their individual abilities, innate needs, and deeply held values [3, 4], has emerged as a promising strategy for enhancing both individual well-being and performance in the nursing profession [5, 6]. Essentially, job crafting empowers employees to take ownership of their work, transforming it from a prescribed set of tasks into a personally meaningful and engaging experience.

Job crafting is typically understood to encompass three distinct dimensions: task crafting, which involves changing the scope or nature of one's responsibilities; cognitive crafting, the process of reframing the perceived meaning and purpose of work; and relational crafting, which involves building and maintaining positive relations with colleagues and clients to foster a more supportive and fulfilling work environment [4, 7]. While other approaches to job crafting exist, such as resource-based crafting (focusing on seeking and utilizing resources [8]) and regulatory-focus-based crafting (focused on promotion or prevention [9]), the present study adopts the role-based approach due to its comprehensive coverage of the various aspects of job crafting that are frequently observed in the nursing context. Task, relational, and cognitive crafting allow for a more granular understanding of how nurses proactively shape their jobs in response to the demands and opportunities within their roles [6, 10]. By engaging in these crafting behaviors, nurses can mitigate the negative effects of workplace stressors, increase their sense of autonomy and control, and ultimately enhance their job satisfaction and commitment [11, 12]. While research on job crafting has proliferated across various occupational settings, the specific factors that drive these behaviors in nurses remain relatively underexplored [13]. Understanding these antecedents is critical to developing targeted interventions to promote job crafting and, in turn, improve nurses' well-being and patient outcomes.

A person's belief in free will (BFW) is a potentially influential psychological factor that is often overlooked. BFW is defined as the conviction that humans have genuine agency and control over their decisions and actions [14, 15]. It reflects the extent to which people see themselves as the authors of their own lives. We chose BFW as a key predictor because of the demanding and ethically complex nature of nursing. Nurses frequently face situations requiring critical decisions, high responsibility, and a strong sense of purpose [16, 17]. In this context, BFW empowers nurses to proactively shape their roles and persevere through challenges. Psychologically, BFW is not just an abstract concept. It is a core belief that shapes motivation, behavior, and personal responsibility [18]. Research links stronger BFW to positive outcomes, such as enhanced goal pursuit, self-control, and work ethic [19, 20]. This is likely because people who believe their choices matter are more likely to invest effort, persist through difficulties, and take ownership of their actions [14, 21].

The relationship between BFW and job crafting can be further understood through the lens of self-determination theory (SDT) [22]. SDT posits that individuals are intrinsically motivated when their needs for autonomy, competence, and relatedness are satisfied. BFW can be seen as a key cognitive foundation for experiencing autonomy, as it reflects the belief that one's actions are self-determined rather than imposed by external forces [14, 18]. When individuals hold a strong BFW, they are more likely to perceive themselves as having control over their work environment, leading to greater intrinsic motivation to proactively craft their jobs in ways that enhance their competence and foster positive relations. This suggests that BFW may serve as a catalyst for self-initiated behaviors aligned with the core tenets of SDT, ultimately promoting job crafting.

In the context of nursing, BFW may play a particularly important role in fostering proactive and adaptive work behaviors. The core argument is that nurses who firmly believe their actions hold significance are more likely to be intrinsically motivated to actively shape their work environment and consciously redefine their roles to better align with their personal values and aspirations [23]. This intrinsic motivation to actively craft one's job is especially relevant in environments like nursing, where workloads and working conditions are difficult to influence [24]. This proactive approach to shaping one's professional life could have a significant impact on whether or not a nurse finds her/his career rewarding. While a growing body of research suggests an association between BFW and various desirable behaviors [19, 20], its specific association with job crafting, particularly in the highly stressful, demanding, and rapidly changing environment of contemporary nursing, warrants more systematic investigation.

It is also crucial to recognize that individual differences in fundamental personality traits may have a significant link with both BFW and the propensity to engage in job crafting [25, 26]. For example, individuals high in conscientiousness and proactivity may be predisposed to BFW and actively seek opportunities to job craft, regardless of external circumstances [27, 28]. Furthermore, it has been reported that the Big Five personalities (neuroticism, conscientiousness, agreeableness, extraversion, and openness) may differentially influence job crafting [29], potentially confounding the relationship between BFW and job crafting. Therefore, when examining the relationship between BFW and job crafting, it is crucial to consider the potential confounding effects of these underlying personality dimensions and control for them statistically, as individual traits such as the Big Five personalities, emotional intelligence, and autonomy could theoretically explain alternative relationships among the studied variables [26, 29–31].

### 1.1. The Present Study

To address these gaps in the existing literature, this study examined the link between BFW and job crafting among nurses, explicitly considering the potential influence of fundamental personality traits. We hypothesized that a stronger endorsement of BFW would be positively associated with greater engagement in job crafting activities. Furthermore, we hypothesized that this association would remain significant even after controlling for the potential confounding effects of the Big Five personality traits [32]. Recognizing the limitations inherent in relying solely on a self-report measure of BFW, and acknowledging that this measure may inadvertently capture constructs beyond pure BFW, we adopted a two-pronged methodological approach to more rigorously assess this association.

In Study 1, we used the well-established free will self-report questionnaire, while simultaneously controlling for the Big Five personality traits to isolate the unique contribution of BFW to job crafting. In Study 2, we used a novel, direct philosophical prompt adapted from the work of Nichols and Knobe [33] to classify participants as either committed free will believers or ardent determinism believers. Particularly, ardent determinism believers are those who strongly subscribe to the philosophical doctrine of determinism, arguing that all events, including human actions and choices, are causally predetermined by prior events and the laws of nature [33]. This perspective stands in stark contrast to free will believers, whose beliefs were measured on a continuum in Study 1. Then, by comparing the job crafting scores of these two distinct groups, we aimed to strengthen the validity and reliability of our findings. This two-pronged approach was deliberately chosen to provide a robust and highly nuanced understanding of the complex role of BFW in shaping nurses' individual experiences of their work.

## 2. Methods

### 2.1. Study 1

#### 2.1.1. Study Design and Participants

In Study 1, we aimed to conduct a preliminary investigation into the hypothesized link between BFW and job crafting. Critically, the study controlled for the influence of the Big Five personality traits, variables documented to share associations with both the BFW and the tendency to engage in job crafting [25, 26], in order to ascertain the unique effect of BFW.

Data were collected from nurses at a hospital in Chengdu, a city in the Sichuan province of Southwest China, using the method of convenience sampling between January and March of 2021. All participants met specific criteria: they possessed a valid nursing license, had at least 1 year of experience in clinical or managerial nursing roles, and reported no prior history of substance dependence or psychiatric illness. Invitations to participate in the survey were disseminated via a dedicated WeChat group for the hospital's nursing staff, with the assistance of hospital administrators. A total of 282 questionnaires were initially collected. However, after data curation, 8 responses were excluded due to issues such as incompleteness, evidence of a lack of effort (e.g., the completion time far below the expected time), or inconsistent responding patterns (e.g., selecting the same response for all items). This resulted in a final sample of 274 completed questionnaires for analysis. During the survey, participants first reported their sociodemographic characteristics, after which they completed a series of standard measures on BFW, job crafting, and Big Five personalities. Notably, the choice of the study measures was dictated by the availability of the validated, culturally appropriate instruments for the Chinese nursing population.

#### 2.1.2. Variables and Measures

*BFW*. The degree to which participants endorsed BFW was evaluated using the Free Will Scale (FWS) from the Free Will Inventory (FWI) [15]. The FWI is designed to measure not only BFW but also related concepts like determinism. The unidimensional FWS consists of five items, each rated on a seven-point Likert scale ranging from “*strongly disagree*” (1) to “*strongly agree*” (7). Higher scores indicate greater BFW. Evidence from Chinese populations suggests that the FWS possesses sufficient reliability and validity, with Cronbach's alpha ranging from 0.71 to 0.75 [34]. For this study, Cronbach's alpha for the FWS was found to be 0.77, indicating a suitable level of internal reliability.

*Job crafting*. Participants' engagement in job crafting activities was assessed using the Measure of Job Crafting (MJC) [7]. This 21-item scale encompasses three subscales: task crafting (e.g., changing tasks for interest), cognitive crafting (e.g., finding purpose in the job), and relational crafting (e.g., building connections with colleagues). A 5-point Likert scale (ranging from 1 = “*strongly disagree*” to 5 = “*strongly agree*”) was used for participants to rate each statement. Elevated scores indicated a higher degree of endorsement for the job crafting-related items, suggesting greater job crafting. The Chinese version of the MJC has demonstrated satisfied reliability and validity in different populations including nurses, with Cronbach's alpha ranging from 0.84 to 0.89 [35, 36]. Notably, we used the MJC to measure job crafting instead of other instruments, such as the Job Crafting Questionnaire (JCQ; [37], for a specific reason. While the JCQ is more commonly used to evaluate the three dimensions of job crafting, a psychometrically sound Chinese version of the JCQ was not available in the literature. However, Zhu [38] translated and validated a Chinese version of the MJC, which is available through the China National Knowledge Infrastructure database. For this study, Cronbach's alpha for the MJC was found to be 0.86, indicating an adequate internal reliability.

*Big Five personalities*. To account for the possible effects of broad personality traits on the link between BFW and job crafting, the Big Five Inventory (BFI) [39] was used. The BFI, a 44-item questionnaire, measures five key personality dimensions: neuroticism, conscientiousness, agreeableness, extraversion, and openness. Participants indicated their self-perceptions on a 5-point Likert scale, from “*not at all like me*” (1) to “*very much like me*” (5). Studies have indicated that the BFI demonstrates sufficient reliability and validity among Chinese populations, specifically including nurses, with Cronbach's alpha ranging from 0.72 to 0.81 [40, 41]. For this study, the BFI subscales showed acceptable internal reliability, as indicated by Cronbach's alpha values ranging from 0.69 to 0.78.

#### 2.1.3. Data Analysis

Statistical analyses were conducted using IBM SPSS Version 25.0 (SPSS Inc., Chicago, IL, USA). Initially, descriptive statistics were computed, and Pearson correlations were examined among all study variables. Then, common method bias, potentially arising from the self-report nature of the questionnaire data, was assessed using Harman's single-factor test [42, 43]. The test involved exploratory factor analysis; the absence of a single factor explaining over 40% of the variance suggested minimal common method bias [42, 43]. Subsequently, hierarchical regression analyses were performed to assess the specific predictive capacity of BFW on job crafting. For these regressions, the MJC total score, along with its subscale scores, served as the dependent variables. In Step 1, demographic variables (age, sex, education level, and working years) and BFI scores were entered as independent variables, followed by FWS scores as independent variables in Step 2.

### 2.2. Study 2

#### 2.2.1. Study Design and Participants

A potential weakness of Study 1 is that the FWS might capture constructs beyond BFW, potentially impacting its relationship with job crafting. For example, an item such as “People ultimately have complete control over their decisions and their actions” bears resemblance to self-control. Therefore, even though Study 1 controlled for the Big Five personality traits, the “unpurified” nature of the FWS leaves open the possibility that the observed link between BFW and job crafting may be attributable to other factors. To mitigate this concern, Study 2 adopted an approach designed by Nichols and Knobe [33] to assess BFW in a more direct and less confounded manner. This method involved presenting participants with two distinct scenarios describing a deterministic universe versus a free will universe. Participants were then asked to identify which universe best reflected their own beliefs. Based on their selections, individuals were categorized as either free will believers or determinism believers. Subsequently, the FWS and MJC scores of these two groups were compared.

Nurses from several hospitals in Chengdu and Kunming, situated in southwest China, were recruited for this study by using the method of convenience sampling between November 2021 and May 2022. With the support of hospital administrators, we disseminated the survey link through WeChat groups created specifically for nurses at each institution. This data collection was part of a larger, ongoing research project aimed at identifying factors that may influence nurses' work performance and well-being [44]. Initially, 446 nurses submitted questionnaires, of which 435 were subsequently deemed valid. The same validity criteria used in Study 1 were applied in Study 2 to ensure data quality.

#### 2.2.2. Variables and Measures

The FWS and MJC were identical to those used in Study 1. The internal consistency of these measures in this study was satisfactory, with Cronbach's alpha values of 0.80 for the FWS and 0.87 for the MJC. These reliability estimates were comparable to those observed in Study 1.

To directly assess BFW, we utilized a single philosophical question developed by Nichols and Knobe [33], a method previously validated with Chinese samples [26, 28]. Participants initially reviewed descriptions of both a deterministic scenario and a free will scenario as follows:

“Imagine a universe (Universe A) in which everything that happens is completely caused by whatever happened before it. This is true from the very beginning of the universe, so what happened in the beginning of the universe caused what happened next, and so on right up until the present. For example, one day. John decided to have French fries at lunch. Like everything else, this decision was completely caused by what happened before it. So, if everything in this universe was exactly the same up until John made his decision, then it had to happen that John would decide to have French fries.”

“Now imagine a universe (Universe B) in which almost everything that happens is completely caused by whatever happened before it. The one exception is human decision making. For example, one day Mary decided to have French fries at lunch. Since a person's decision in this universe is not completely caused by what happened before it, even if everything in the universe was exactly the same up until Mary made her decision, it did not have to happen that Mary would decide to have French fries. She could have decided to have something different.”

Following scenario review, participants were asked to select the universe that most closely resembled their own. Those who chose Universe A were classified as determinism believers, while all others were designated as free will believers.

#### 2.2.3. Data Analysis

Similar to Study 1, descriptive statistics, Pearson correlation analyses, and Harman's single-factor test on common method bias were performed in Study 2. Furthermore, independent samples *t*-tests were used to explore any psychological differences (e.g., the FWS and MJC scores) between participants who believe in free will and those who believe in determinism.

### 2.3. Ethics Statement

The Ethics Committee of West China Hospital of Sichuan University approved the above two studies (approval number: 2020HXBH092). All participants received detailed information regarding the study's purpose, procedures, potential benefits, and possible risks before providing informed consent. Participation was entirely voluntary, and participants were informed of their right to withdraw at any time without penalty. Data were collected anonymously, and no identifying information was linked to the responses. Confidentiality was strictly maintained by storing data securely and using aggregated results for reporting.

## 3. Results

### 3.1. Study 1

#### 3.1.1. Descriptive Statistics, Correlation Analyses, and Common Method Bias

The study sample, as detailed in Table 1, comprised 274 nurses aged 20–53 years, with a significant majority being female (96.3%). Key demographic features included that over half were married (69.3%), held a primary title (50.7%), and had attained a bachelor's degree (54.7%). Years of experience in nursing varied from 1 to 36 years across the participants.

Descriptive statistics (means and standard deviations) and bivariate correlations for all study variables are presented in Table 2. With the exception of the relationship between BFW and neuroticism as measured by the BFI, all bivariate correlations achieved statistical significance at *p* < 0.01. Critically, BFW displayed positive correlations with job crafting and its three dimensions (rs > 0.18, ps < 0.01).

Examination for common method bias showed 19 eigenvalues exceeding 1. The first eigenvalue accounted for only 16.67% of the variance, well below the 40% threshold, suggesting no substantial common method bias in the study data [42, 43].

#### 3.1.2. Hierarchical Regression Analyses

To determine if the association between BFW and job crafting remained significant after adjusting for potential confounding variables, specifically the Big Five personality traits, hierarchical regression analyses were conducted. The regression models incorporated demographic variables (age, sex, education level, and working years) and BFI personality dimensions in the first step, followed by BFW in the second step. The analyses indicated that BFW significantly predicted job crafting (*β* = 0.11, *p* < 0.05) and its task (*β* = 0.11, *p* < 0.05) and cognitive (*β* = 0.12, *p* < 0.05) dimensions, even after controlling for the Big Five personalities and demographic variables. However, BFW did not significantly predict relational crafting (*β* = 0.06, *p* > 0.05) beyond the influence of the Big Five personalities and demographic variables (Table 3).

### 3.2. Study 2

#### 3.2.1. Descriptive Statistics, Correlation Analyses, and Common Method Bias

As shown in Table 1, this study included 435 nurses with demographic characteristics mirroring those in Study 1. The majority were female (87.1%), between 18 and 55 years old, married (66.7%), held a primary professional title (52.2%), and possessed a bachelor's degree (57.0%). Nursing experience ranged from 1 to 39 years.

In an effort to replicate the primary findings of Study 1, we examined the correlation between scores on the FWS and the MJC. As anticipated, the results mirrored those of the previous study. Specifically, BFW, as measured by the FWS, was positively correlated with overall job crafting (*r* = 0.29, *p* < 0.01) and each of its dimensions: task crafting (*r* = 0.29, *p* < 0.01), cognitive crafting (*r* = 0.27, *p* < 0.01), and relational crafting (*r* = 0.23, *p* < 0.01).

The common method bias testing yielded five eigenvalues greater than 1. The first factor accounted for 25.13% of the variance, which is less than the 40% threshold, thus indicating the absence of significant common method bias [42, 43].

#### 3.2.2. Comparisons Between Free Will Believers and Determinism Believers

Similar to earlier research [26, 28, 33], the majority of participants (*N* = 336, 77.2%) selected Universe B, representing the free will scenario. Subsequently, we compared the FWS scores between the two groups. As indicated in Table 4, free will believers exhibited significantly higher scores on the FWS than determinism believers (*t*[433] = 2.20, *p* < 0.05). These findings support the convergent validity of the two measures of BFW.

Most importantly, and in line with our hypothesis, we compared scores on the MJC between free will and determinism believers (Table 4). As expected, free will believers reported significantly higher levels of overall job crafting (*t*[433] = 2.81, *p* < 0.01) compared to determinism believers. This pattern extended to each dimension of job crafting: task crafting (*t*[433] = 2.01, *p* < 0.05), cognitive crafting (*t*[433] = 2.92, *p* < 0.01), and relational crafting (*t*[433] = 2.67, *p* < 0.01).

## 4. Discussion

This research aimed to investigate the relationship between nurses' BFW and their engagement in job crafting. Across two distinct studies, using both correlational and group comparison methods, our findings consistently supported the hypothesis that a stronger endorsement of the BFW is positively associated with greater job crafting in nurses. Specifically, Study 1 demonstrated that the BFW significantly predicted overall job crafting, as well as task and cognitive crafting dimensions, even after controlling for the Big Five personality traits. Study 2 provided convergent evidence, revealing that nurses who identified themselves as free will believers reported significantly higher levels of job crafting than those who endorsed a deterministic worldview. These findings make a meaningful contribution to the growing literature on job crafting and highlight the potential importance of nurses' fundamental beliefs about agency and control in shaping their work experiences.

This study contributes to the job crafting literature in several key ways. First, it extends the understanding of the antecedents of job crafting by demonstrating the significant role of BFW, a previously underexplored psychological factor. While existing research has primarily focused on personality traits, job characteristics, and organizational contexts [45, 46], our findings highlight the importance of individuals' core beliefs about their own agency in shaping their work experiences. Second, this research provides empirical evidence for the applicability of SDT to the understanding of job crafting. By showing that BFW, a key foundation for experiencing autonomy [18], is positively associated with job crafting, we provide support for the SDT-based argument that individuals are more likely to proactively shape their jobs when they perceive themselves as having control over their work environment [22]. Finally, the use of a two-pronged methodological approach, combining a self-report scale with a direct philosophical prompt, strengthens the validity and reliability of our findings, providing a more robust assessment of the link between BFW and job crafting.

The observed positive link between BFW and job crafting aligns with theoretical frameworks suggesting that individuals who perceive themselves as autonomous agents are more likely to take proactive steps to shape their environment and create a sense of purpose and fulfillment in their work [47, 48]. Nurses who believe their actions can make a difference may be more motivated to actively seek opportunities to modify their tasks, reframe the cognitive meaning of their work, and cultivate supportive relationships with colleagues and patients, leading to higher levels of job crafting. This is particularly relevant in the demanding and often inflexible context of nursing, where intrinsic motivation and self-initiated adaptations may be critical for maintaining engagement and preventing burnout [49, 50].

Furthermore, the finding in Study 1 that BFW specifically predicted task and cognitive crafting, but not relational crafting, after adjusting for Big Five personality traits warrant further consideration. The nonsignificant finding for relational crafting in Study 1 suggests that the relationship between BFW and relational crafting is more complex than initially hypothesized. While BFW may foster a general sense of agency and motivation to shape one's work, its impact on relational crafting appears to be less direct and potentially mediated by other factors, such as personality traits. This is generally consistent with prior research suggesting that personality plays a role in social aspects of job crafting [25, 29]. For example, individuals high in extraversion and agreeableness and low in neuroticism are often more inclined to engage in relational crafting activities (e.g., building positive relationships with colleagues and patients) due to their inherent social skills and tendencies [25, 29, 39]. The lack of a significant incremental effect of BFW on relational crafting beyond Big Five personalities indicates that while BFW can increase a nurse's overall effort and perceived impact at work, it does not necessarily translate into more relational crafting. To increase relational crafting, nurses must not only believe in the utility of relation-based adjustments but also feel able to successfully make those adjustments, and those feelings are closely related to the Big Five personalities [51]. It is possible that relational crafting is influenced by a more complex interplay of factors, including personality traits as well as contextual factors such as team dynamics and organizational culture [52, 53]. The limited correlation between BFW and relational crafting may be because BFW does not directly produce the types of social acumen and stable attitudes found to promote strong interpersonal bonds [54]. Although BFW did not have an incremental effect on relational crafting beyond Big Five personalities, it also did not have a negative effect. This suggests that as the industry changes, BFW may begin to exhibit a stronger effect as nurses take on more responsibility and can more actively influence the work.

The use of two distinct methodological approaches to assess BFW—a self-report scale in Study 1 and a direct philosophical prompt in Study 2—strengthens the validity and reliability of our findings. The fact that both approaches yielded consistent results supports the robustness of the link between BFW and job crafting. The convergent validity of these measures is further supported by the finding that nurses who chose the free will scenario in Study 2 also scored significantly higher on the FWS, demonstrating a consistency between their explicit philosophical beliefs and their implicit sense of personal agency, which fits with the similar findings in other Chinese populations [26, 28]. In a sense, these findings suggest that nurses understand their job to be dynamic and influenced by their own efforts.

This study has several limitations. First, the cross-sectional nature of the studies precludes definitive conclusions about causality. While our findings suggest that BFW is associated with job crafting, it is possible that the relationship is reciprocal or that other unmeasured factors drive both variables. Future longitudinal research is needed to examine the temporal sequence of BFW and job crafting and to explore potential mediating or moderating mechanisms. Second, the studies were conducted with nurses in China, which limits the generalizability of the findings to other cultural contexts. Future research should examine the relationship between BFW and job crafting in diverse nursing populations, taking into account potential cultural differences in beliefs about agency, work values, and organizational practices [55]. Third, the use of self-report measure of job crafting may be subject to social desirability bias [56], in which participants may over-report job crafting to present themselves in a more favorable light. Although steps were taken to ensure participant anonymity and confidentiality, future research could consider using more objective measures of job crafting, such as observational data or supervisor ratings. Finally, classifying participants into binary groups (free will vs. determinism believers) in Study 2 based on a single philosophical prompt may oversimplify the complexity and nuances of their actual beliefs regarding free will and determinism. While this approach allowed for a more direct assessment of BFW, it may have resulted in an artificial categorization of individuals who hold more nuanced or mixed views [28]. It is possible that some participants who identified with the free will scenario may still acknowledge the influence of determinism on their decisions, while those who chose the deterministic scenario may not entirely deny the possibility of free will. This limitation should be considered when interpreting the findings of Study 2, and future research could explore alternative methods for assessing BFW that capture the full spectrum of beliefs.

### 4.1. Implications for Nursing Management and Practice

The current findings have several practical implications for nursing management. First, they suggest that fostering a work environment that promotes a sense of autonomy, control, and personal meaning may be particularly effective in promoting job crafting among nurses. Strategies such as shared governance models, participatory decision-making processes, and professional development opportunities can empower nurses to take ownership of their work and actively shape their roles [57, 58]. By providing nurses with the freedom and resources to shape their work, healthcare organizations can potentially improve nurses' well-being, increase their job satisfaction, and improve the quality of patient care. Second, given the observed link of BFW with job crafting, it may be worthwhile to explore interventions specifically designed to cultivate a stronger sense of personal agency and BFW among nurses. While it may be difficult to directly manipulate philosophical beliefs, interventions that focus on enhancing self-efficacy, promoting goal-setting skills, and fostering a sense of purpose and meaning in work may indirectly strengthen nurses' BFW and, consequently, their propensity to engage in job crafting activities [19, 28]. Third, our findings highlight the importance of considering basic personality traits when designing and implementing job crafting interventions. Although BFW appears to be a significant predictor of job crafting, its influence is likely intertwined with other personality dimensions [25, 26, 52]. Nurse managers should be mindful of these factors and tailor their approaches to meet the specific needs and preferences of their staff. By recognizing and leveraging the unique strengths and attributes of each nurse, leaders can create a work environment that fosters both individual well-being and collective success.

## 5. Conclusion

In conclusion, this research provides compelling evidence for a positive association between nurses' BFW and their engagement in job crafting activities. By highlighting the importance of this often overlooked psychological factor, our findings contribute to a deeper understanding of the individual and contextual factors that shape nurses' work experiences. As healthcare systems continue to evolve and face increasing demands, fostering a work environment that empowers nurses to take ownership of their jobs and actively craft their roles may be essential for promoting nurses' well-being, enhancing their engagement, and ultimately improving the quality of patient care.

## Figures and Tables

**Table 1:** Sociodemographic characteristics of the participants.

Measure	Mean ± SD (range) *N*%	
	Study 1	Study 2
Sex		
Female	264 (96.3%)	379 (87.1%)
Male	10 (3.7%)	56 (12.9%)
Age (years)	32.20 ± 7.67 (20–53)	31.46 ± 7.19 (18–55)
Education level		
Graduate degree	0 (0.0%)	4 (0.9%)
Bachelor degree	150 (54.7%)	248 (57.0%)
College degree	111 (40.5%)	159 (36.6%)
Secondary vocational degree	13 (4.8%)	24 (5.5%)
Marital status		
Married	190 (69.3%)	290 (66.7%)
Unmarried	76 (27.7%)	122 (28.0%)
Divorced	6 (2.2%)	20 (4.6%)
Widowed	2 (0.8%)	3 (0.7%)
Professional title		
Vice senior	2 (0.7%)	8 (1.8%)
Intermediate	75 (27.4%)	95 (21.9%)
Primary	139 (50.7%)	227 (52.2%)
None	58 (21.2%)	105 (24.1%)
Length of nursing work (years)	11.35 ± 8.47 (1–36)	10.24 ± 7.88 (1–39)

Abbreviations: *N*, number; SD, standard deviation.

**Table 2:** Descriptive statistics and bivariate correlations in Study 1.

Measure	Mean ± SD	1	2	3	4	5	6	7	8	9
1. FWS	3.89 ± 0.50	—								
2. MJC	3.29 ± 0.47	0.26^∗∗^	—							
3. MJC-T	3.36 ± 0.53	0.25^∗∗^	0.89^∗∗^	—						
4. MJC-C	3.39 ± 0.52	0.26^∗∗^	0.89^∗∗^	0.71^∗∗^	—					
5. MJC-R	3.13 ± 0.54	0.18^∗∗^	0.88^∗∗^	0.67^∗∗^	0.67^∗∗^	—				
6. BFI-E	3.14 ± 0.53	0.16^∗∗^	0.27^∗∗^	0.26^∗∗^	0.21^∗∗^	0.24^∗∗^	—			
7. BFI-A	3.89 ± 0.42	0.25^∗∗^	0.50^∗∗^	0.47^∗∗^	0.50^∗∗^	0.37^∗∗^	0.20^∗∗^	—		
8. BFI-C	3.62 ± 0.50	0.16^∗∗^	0.65^∗∗^	0.59^∗∗^	0.58^∗∗^	0.55^∗∗^	0.30^∗∗^	0.46^∗∗^	—	
9. BFI-N	2.92 ± 0.60	−0.10	−0.50^∗∗^	−0.44^∗∗^	−0.42^∗∗^	−0.48^∗∗^	−0.46^∗∗^	−0.43^∗∗^	−0.50^∗∗^	—
10. BFI-O	3.17 ± 0.48	0.17^∗∗^	0.34^∗∗^	0.31^∗∗^	0.28^∗∗^	0.31^∗∗^	0.37^∗∗^	0.20^∗∗^	0.30^∗∗^	−0.31^∗∗^

Abbreviations: BFI-A, agreeableness subscale of Big Five Inventory; BFI-C, conscientiousness subscale of Big Five Inventory; BFI-E, extraversion subscale of Big Five Inventory; BFI-N, neuroticism subscale of Big Five Inventory; BFI-O, openness subscale of Big Five Inventory; FWS, Free Will Scale; MJC, measure of job crafting; MJC-C, cognitive crafting subscale of MJC; MJC-R, relational crafting subscale of MJC; MJC-T, task crafting subscale of MJC; SD, standard deviation.

^∗^ *p* < 0.05.

^∗∗^ *p* < 0.01.

**Table 3:** Hierarchical regression models for job crafting in Study 1.

	MJC		MJC-T		MJC-C		MJC-R	
	*B* (SE)	*β*	*B* (SE)	*β*	*B* (SE)	*β*	*B* (SE)	*β*
*Step 1*								
Age (years)	0.12 (0.21)	0.09	0.11 (0.08)	0.23	0.05 (0.08)	0.11	−0.05 (0.09)	−0.09
Sex^a^	4.24 (2.30)	0.08	2.04 (0.93)	0.10^∗^	0.84 (0.92)	0.04	1.36 (1.00)	0.07
Education years	−1.19 (0.74)	−0.07	−0.24 (0.30)	−0.04	−0.64 (0.29)	−0.10^∗^	−0.31 (0.32)	−0.05
Working years	−0.19 (0.19)	−0.16	−0.13 (0.08)	−0.29	−0.06 (0.07)	−0.14	0.00 (0.08)	0.00
BFI-E	−0.09 (0.12)	−0.04	0.01 (0.05)	0.01	−0.04 (0.05)	−0.05	−0.06 (0.05)	−0.07
BFI-A	0.52 (0.13)	0.20^∗∗^	0.20 (0.05)	0.21^∗∗^	0.25 (0.05)	0.26^∗∗^	0.07 (0.06)	0.07
BFI-C	1.01 (0.12)	0.46^∗∗^	0.35 (0.05)	0.43^∗∗^	0.32 (0.05)	0.39^∗∗^	0.34 (0.05)	0.40^∗∗^
BFI-N	−0.37 (0.11)	−0.18^∗∗^	−0.08 (0.05)	−0.11	−0.09 (0.05)	−0.12	−0.20 (0.05)	−0.25^∗∗^
BFI-O	0.25 (0.10)	0.12^∗^	0.08 (0.04)	0.10^∗^	0.07 (0.04)	0.10	0.10 (0.04)	0.12^∗^

*Step 2*								
Age (years)	0.14 (0.20)	0.11	0.12 (0.08)	0.25	0.06 (0.08)	0.13	−0.04 (0.09)	−0.08
Sex^a^	4.09 (2.29)	0.08	1.98 (0.92)	0.10^∗^	0.78 (0.91)	0.04	1.33 (1.00)	0.07
Education years	−1.12 (0.73)	−0.07	−0.21 (0.30)	−0.03	−0.61 (0.29)	−0.10^∗^	−0.30 (0.32)	−0.05
Working years	−0.20 (0.18)	−0.17	−0.13 (0.07)	−0.30	−0.06 (0.07)	−0.15	−0.00 (0.08)	−0.00
BFI-E	−0.11 (0.12)	−0.05	0.00 (0.05)	0.00	−0.05 (0.05)	−0.06	−0.06 (0.05)	−0.07
BFI-A	0.46 (0.13)	0.18^∗∗^	0.18 (0.05)	0.19^∗∗^	0.22 (0.05)	0.24^∗∗^	0.06 (0.06)	0.06
BFI-C	1.00 (0.12)	0.45^∗∗^	0.35 (0.05)	0.42^∗∗^	0.31 (0.05)	0.38^∗∗^	0.34 (0.05)	0.40^∗∗^
BFI-N	−0.39 (0.11)	−0.19^∗∗^	−0.09 (0.05)	−0.12^∗^	−0.10 (0.05)	−0.13^∗^	−0.20 (0.05)	−0.26^∗∗^
BFI-O	0.23 (0.10)	0.11^∗^	0.07 (0.04)	0.09	0.06 (0.04)	0.08	0.09 (0.04)	0.12^∗^
FWS	0.42 (0.18)	0.11^∗^	0.16 (0.07)	0.11^∗^	0.18 (0.07)	0.12^∗^	0.08 (0.08)	0.06

Abbreviations: BFI-A, agreeableness subscale of Big Five Inventory; BFI-C, conscientiousness subscale of Big Five Inventory; BFI-E, extraversion subscale of Big Five Inventory; BFI-N, neuroticism subscale of Big Five Inventory; BFI-O, openness subscale of Big Five Inventory; FWS, Free Will Scale; MJC, Measure of Job Crafting; MJC-C, cognitive crafting subscale of MJC; MJC-R, relational crafting subscale of MJC; MJC-T, task crafting subscale of MJC; SE, standard error.

^a^Male = 0, female = 1.

^∗^ *p* < 0.05.

^∗∗^ *p* < 0.01.

**Table 4:** Psychological differences between free will and determinism believers in Study 2.

Measure	Free will believers (*N* = 336)	Determinism believers (*N* = 99)	*t*	*p*
FWS	3.90 ± 0.53	3.76 ± 0.56	2.20	0.030
MJC	3.38 ± 0.48	3.23 ± 0.45	2.81	0.006
MJC-T	3.43 ± 0.53	3.31 ± 0.50	2.01	0.046
MJC-C	3.49 ± 0.52	3.32 ± 0.47	2.92	0.004
MJC-R	3.23 ± 0.53	3.06 ± 0.54	2.67	0.008

*Note:* Data are presented as mean ± standard deviation.

Abbreviations: FWS, Free Will Scale; MJC, measure of job crafting; MJC-C, cognitive crafting subscale of MJC; MJC-R, relational crafting subscale of MJC; MJC-T, task crafting subscale of MJC; *N*, number.

## Data Availability

The data that support the findings of this study are available from the corresponding author upon reasonable request.

## References

[1] Mohammadnejad F., Freeman S., Klassen-Ross T., Hemingway D., Banner D. (2023). Impacts of Technology Use on the Workload of Registered Nurses: A Scoping Review. *Journal of Rehabilitation and Assistive Technologies Engineering*.

[2] Tamata A. T., Mohammadnezhad M. (2023). A Systematic Review Study on the Factors Affecting Shortage of Nursing Workforce in the Hospitals. *Nursing Open*.

[3] Demerouti E. (2014). Design Your Own Job Through Job Crafting. *European Psychologist*.

[4] Wrzesniewski A., Dutton J. E. (2001). Crafting a Job: Revisioning Employees as Active Crafters of Their Work. *Academy of Management Review*.

[5] Gordon H. J., Demerouti E., Le Blanc P. M., Bakker A. B., Bipp T., Verhagen M. A. M. T. (2018). Individual Job Redesign: Job Crafting Interventions in Healthcare. *Journal of Vocational Behavior*.

[6] Han S. (2023). Nurses’ Job Crafting, Work Engagement, and Well-Being: A Path Analysis. *BMC Nursing*.

[7] Dvorak K. (2014). *The Theoretical Development and Empirical Testing of the Measure of Job Crafting (mjc)*.

[8] Tims M., Bakker A. B. (2010). Job Crafting: Towards a New Model of Individual Job Redesign. *SA Journal of Industrial Psychology*.

[9] Lichtenthaler P. W., Fischbach A. (2019). A Meta-Analysis on Promotion-and Prevention-Focused Job Crafting. *European Journal of Work & Organizational Psychology*.

[10] Harbridge R., Ivanitskaya L., Spreitzer G., Boscart V. (2022). Job Crafting in Registered Nurses Working in Public Health: A Qualitative Study. *Applied Nursing Research*.

[11] El-Gazar H. E., Abdelhafez S., Ibrahim N., Shawer M., Zoromba M. A. (2023). Effect of Job Crafting Intervention Program on Harmonious Work Passion and Career Commitment Among Nurses: A Randomized Controlled Trial. *Journal of Nursing Management*.

[12] Li J. Y., Yang H., Weng Q. X., Zhu L. N. (2023). How Different Forms of Job Crafting Relate to Job Satisfaction: The Role of Person-Job Fit and Age. *Current Psychology*.

[13] Wang Y., Yang Q. F., Wang L. W., Zhang Q. W., Li Y. L. (2024). The Factors of Job Crafting in Emergency Nurses: Regression Models Versus Qualitative Comparative Analysis. *BMC Nursing*.

[14] Feldman G. (2017). Making Sense of Agency: Belief in Free Will as a Unique and Important Construct. *Social and Personality Psychology Compass*.

[15] Nadelhoffer T., Shepard J., Nahmias E., Sripada C., Ross L. T. (2014). The Free Will Inventory: Measuring Beliefs About Agency and Responsibility. *Consciousness and Cognition*.

[16] Huffman D. M., Rittenmeyer L. (2012). How Professional Nurses Working in Hospital Environments Experience Moral Distress: A Systematic Review. *Critical Care Nursing Clinics of North America*.

[17] Sperling D. (2021). Ethical Dilemmas, Perceived Risk, and Motivation Among Nurses During the COVID-19 Pandemic. *Nursing Ethics*.

[18] Baumeister R. F. (2008). Free Will in Scientific Psychology. *Perspectives on Psychological Science*.

[19] Crescioni A. W., Baumeister R. F., Ainsworth S. E., Ent M., Lambert N. M. (2016). Subjective Correlates and Consequences of Belief in Free Will. *Philosophical Psychology*.

[20] Genschow O., Cracco E., Schneider J. (2023). Manipulating Belief in Free Will and Its Downstream Consequences: A Meta-Analysis. *Personality and Social Psychology Review*.

[21] Clark C. J., Winegard B. M., Shariff A. F. (2021). Motivated Free Will Belief: The Theory, New (Preregistered) Studies, and Three Meta-Analyses. *Journal of Experimental Psychology: General*.

[22] Deci E. L., Olafsen A. H., Ryan R. M. (2017). Self-Determination Theory in Work Organizations: The State of a Science. *Annual Review of Organizational Psychology and Organizational Behavior*.

[23] Barrick M. R., Mount M. K., Li N. (2013). The Theory of Purposeful Work Behavior: The Role of Personality, Higher-Order Goals, and Job Characteristics. *Academy of Management Review*.

[24] Neill D. (2011). Nursing Workload and the Changing Health Care Environment: A Review of the Literature. *Administrative Issues Journal*.

[25] Bell C., Njoli N. (2016). The Role of Big Five Factors on Predicting Job Crafting Propensities Amongst Administrative Employees in a South African Tertiary Institution. *SA Journal of Human Resource Management*.

[26] Li C. K., Wang S., Zhao Y. J., Kong F., Li J. G. (2016). The Freedom to Pursue Happiness: Belief in Free Will Predicts Life Satisfaction and Positive Affect Among Chinese Adolescents. *Frontiers in Psychology*.

[27] Bakker A. B., Tims M., Derks D. (2012). Proactive Personality and Job Performance: The Role of Job Crafting and Work Engagement. *Human Relations*.

[28] Li J. G., Zhao Y. J., Lin L., Chen J., Wang S. (2018). The Freedom to Persist: Belief in Free Will Predicts Perseverance for Long-Term Goals Among Chinese Adolescents. *Personality and Individual Differences*.

[29] Sameer S. K., Priyadarshi P. (2021). Role of Big Five Personality Traits in Regulatory-Focused Job Crafting. *South Asian Journal of Business Studies*.

[30] Sameer S. K., Priyadarshi P. (2023). How Emotionally Intelligent Employees Manage Their Internal Employability Through Role-Based Job Crafting?–Evidence From Public Sector Enterprises. *International Review of Psycho-Analysis*.

[31] Zhang F., Tims M., Parker S. K. (2025). Combinations of Approach and Avoidance Crafting Matter: Linking Job Crafting Profiles With Proactive Personality, Autonomy, Work Engagement, and Performance. *Journal of Organizational Behavior*.

[32] Costa P. T., McCrae R. R. (1999). A Five-Factor Theory of Personality. *Handbook of Personality: Theory and Research*.

[33] Nichols S., Knobe J. (2007). Moral Responsibility and Determinism: The Cognitive Science of Folk Intuitions. *Noûs*.

[34] Zhao M., Liu J., Huo Y. Q. (2024). The Value of Believing in Free Will: A Prediction on Seeking and Experiencing Meaning in Life. *Applied Psychology-Health and Well Being*.

[35] Li M. L., Sun H. M., Zhou J. H. (2023). The Association Between Character Strengths and Job Crafting Among Nurses in Tertiary Hospitals: A Cross-Sectional Survey. *Nursing Open*.

[36] Yang R., Ming Y., Ma J. H., Huo R. M. (2017). How Do Servant Leaders Promote Engagement? A Bottom-Up Perspective of Job Crafting. *Social Behavior and Personality: An International Journal*.

[37] Slemp G., Vella-Brodrick D. (2013). The Job Crafting Questionnaire: A New Scale to Measure the Extent to Which Employees Engage in Job Crafting. *International Journal of Wellbeing*.

[38] Zhu S. X. (2019). *The Organizational Investment to Improve Nurses’ Work Engagement: Based on Job Crafting*.

[39] Carciofo R., Yang J. Y., Song N., Du F., Zhang K. (2016). Psychometric Evaluation of Chinese-Language 44-Item and 10-Item Big Five Personality Inventories, Including Correlations With Chronotype, Mindfulness and Mind Wandering. *PLoS One*.

[40] Liu X., Li J., Wang S. (2024). The Impact of Grit on Nurses’ Job Performance: Evaluating Chained Mediation Through Perceived Social Support and Self-Esteem. *Journal of Nursing Management*.

[41] Lu M. X., Zhang F., Tang X. H. (2022). Do Type A Personality and Neuroticism Moderate the Relationships of Occupational Stressors, Job Satisfaction and Burnout Among Chinese Older Nurses? A Cross-Sectional Survey. *BMC Nursing*.

[42] Bozionelos N., Simmering M. J. (2022). Methodological Threat or Myth? Evaluating the Current State of Evidence on Common Method Variance in Human Resource Management Research. *Human Resource Management Journal*.

[43] Gorrell G., Ford N., Madden A., Holdridge P., Eaglestone B. (2011). Countering Method Bias in Questionnaire-Based User Studies. *Journal of Documentation*.

[44] Zhang X. Y., Qiu C., Li X. L., Shekara A., Suo X. L., Wang S. (2025). Examining the Mediating Role of Grit and Self-Efficacy in the Association Between Growth Mindset and Job Satisfaction in a Sample of Chinese Nurses. *Journal of Nursing Management*.

[45] Park S., Park S. (2023). Contextual Antecedents of Job Crafting: Review and Future Research Agenda. *European Journal of Training and Development*.

[46] Petrou P., Demerouti E., Schaufeli W. B. (2015). Job Crafting in Changing Organizations: Antecedents and Implications for Exhaustion and Performance. *Journal of Occupational Health Psychology*.

[47] Feldman G., Farh J. L., Wong K. F. E. (2018). Agency Beliefs Over Time and Across Cultures: Free Will Beliefs Predict Higher Job Satisfaction. *Personality and Social Psychology Bulletin*.

[48] Stillman T. F., Baumeister R. F., Vohs K. D., Lambert N. M., Fincham F. D., Brewer L. E. (2010). Personal Philosophy and Personnel Achievement: Belief in Free Will Predicts Better Job Performance. *Social Psychological and Personality Science*.

[49] Badran F., Akeel A. F. (2020). Job Crafting and Adaptive Performance Among Staff Nurses. *International Journal of Novel Research in Healthcare and Nursing*.

[50] Kohnen D., De Witte H., Schaufeli W. B., Dello S., Bruyneel L., Sermeus W. (2023). What Makes Nurses Flourish at Work? How the Perceived Clinical Work Environment Relates to Nurse Motivation and Well-Being: A Cross-Sectional Study. *International Journal of Nursing Studies*.

[51] Agha Mohammad Hasani P., Mokhtaree M., Sheikh Fathollahi M., Farrokjzadian J. (2018). Interpersonal Communication Skills and Its Association With Personality Dimensions of Nurses in Rafsanjan University of Medical Sciences, Iran, in 2015. *Journal of Occupational Health and Epidemiology*.

[52] Peral S. L., Geldenhuys M. (2020). The Indirect Relationship Between Personality and Performance Through Job Crafting Behaviour. *SA Journal of Industrial Psychology*.

[53] Kim H., Im J., Qu H. L. (2018). Exploring Antecedents and Consequences of Job Crafting. *International Journal of Hospitality Management*.

[54] Rockmann K. W., Bartel C. A. (2025). Interpersonal Relationships in Organizations: Building Better Pipes and Looking Through Prisms. *Annual Review of Organizational Psychology and Organizational Behavior*.

[55] Hopkins B. (2016). *Cultural Differences and Improving Performance: How Values and Beliefs Influence Organizational Performance*.

[56] van de Mortel T. F. (2008). Faking It: Social Desirability Response Bias in Self-Report Research. *Australian Journal of Advanced Nursing*.

[57] Kutney-Lee A., Germack H., Hatfield L. (2016). Nurse Engagement in Shared Governance and Patient and Nurse Outcomes. *The Journal of Nursing Administration: The Journal of Nursing Administration*.

[58] Weston M. J. (2010). Strategies for Enhancing Autonomy and Control Over Nursing Practice. *OJIN: Online Journal of Issues in Nursing*.

